# Molecular cloning, subcellular localization, and rapid recruitment to DNA damage sites of chicken *Ku70*

**DOI:** 10.1038/s41598-024-51501-0

**Published:** 2024-01-12

**Authors:** Manabu Koike, Hideji Yamashita, Yasutomo Yutoku, Aki Koike

**Affiliations:** 1 Institute for Quantum Medical Science, National Institutes for Quantum Science and Technology, 4-9-1 Anagawa, Inage-ku, Chiba, 263-8555 Japan; 2 Life Science Course, Graduate School of Science and Engineering, Saitama University, 255 Shimo-Okubo, Sakura-ku, Saitama, Saitama 338-8570 Japan; 3 Department of Food and Life Sciences, School of Agriculture, Tokai University, 9-1-1 Toroku, Higashi-ku, Kumamoto, 862-8652 Japan

**Keywords:** DNA damage and repair, Biological models

## Abstract

Ku70 is a multifunctional protein with pivotal roles in DNA repair via non-homologous end-joining, V(D)J recombination, telomere maintenance, and neuronal apoptosis control. Nonetheless, its regulatory mechanisms remain elusive. Chicken *Ku70* (*GdKu70*) cDNA has been previously cloned, and DT40 cells expressing it have significantly contributed to critical biological discoveries. GdKu70 features an additional 18 amino acids at its N-terminus compared to mammalian Ku70, the biological significance of which remains uncertain. Here, we show that the 5′ flanking sequence of *GdKu70* cDNA is not nearly encoded in the chicken genome. Notably, these 18 amino acids result from fusion events involving the *NFE2L1* gene on chromosome 27 and the *Ku70* gene on chromosome 1. Through experiments using newly cloned chicken *Ku70* cDNA and specific antibodies, we demonstrated that Ku70 localizes within the cell nucleus as a heterodimer with Ku80 and promptly accumulates at DNA damage sites following injury. This suggests that the functions and spatiotemporal regulatory mechanisms of Ku70 in chickens closely resemble those in mammals. The insights and resources acquired will contribute to elucidate the various mechanisms by which Ku functions. Meanwhile, caution is advised when interpreting the previous numerous key studies that relied on *GdKu70* cDNA and its expressing cells.

## Introduction

Chickens have played a significant role in life science and medical research, serving not only as a nutritional resource but also subjects of study. They have made substantial contributions to drug discovery and fundamental medical research, including vaccine development and the discovery of oncogenes and proto-oncogenes, such as v-src and c-src^1–3^. Over the past 25 years, DT40 cells, derived from chicken B cells, have emerged as valuable models for functional gene studies, significantly advancing in life science research, including basic medical research concerning DNA repair, owing to their high homologous recombination (HR) activity and utility in establishing gene-targeted cell lines^4–14^.

DNA double-strand breaks (DSBs) induced by therapeutic ionizing radiation or heavy particle radiation pose severe threats to vertebrate cells, potentially leading to carcinogenesis, cell death, and cellular senescence^15–18^. Two primary DNA repair pathways exist for DSB: Rad54-dependent HR and Ku (a heterodimer of Ku70 and Ku80)-dependent non-homologous end-joining (NHEJ)^4,5^. In mammalian cells, including humans and mice, NHEJ is the predominant DSB repair mechanism^15–20^. NHEJ initiates with Ku binding to DSB ends^15–18^. Then, DNA-PKcs is recruited there, and XRCC4 and XRCC4-like factor (XLF, also known as NHEJ1 or Cernunnos) promote the ligase activity of DNA ligase IV to rejoin DSB ends^15–18,21,22^. In birds, initial cloning and characterization of chicken *Ku70* (referred to as *GdKu70*) cDNA by Takata et al. revealed distinct features (DDBJ/EMBL/GenBank accession No. AB016529)^5^. By generating Rad54-knockout (KO), Ku70-KO, and Rad54/Ku70-double KO (DKO) DT40 cells, they demonstrated that *GdKu70* cDNA could partially compensate for radiosensitivity when introduced into Rad54/Ku70 DKO-DT40 cells. They also proposed that the predominant repair pathway depends on the cell cycle. Since then, *GdKu70* cDNA, its expressing cells, and derivatives have been instrumental in numerous studies across various life sciences, including radiation biology and DNA repair^4–14^.

*Ku70* cDNA has also been cloned in humans and other organisms, such as mice and dogs^5,23–25^. Chromosomal mapping has shown conserved linkage homology in species like chicken, human, mouse, rat, and hamster^26–29^. GdKu70 contains an additional 18 amino acids at the N-terminal portion compared to human and mouse Ku70^5^. This extra segment is also absent in canine Ku70, suggesting its absence is common among mammalian species^25^. However, the biological significance of this avian-specific N-terminal portion remains uncertain. Understanding its role may shed light on why DT40 cells exhibit high HR activity and uncover avian-specific Ku functions.

In the present study, we cloned chicken *Ku70* cDNA to explore the function and biological significance of the N-terminal-specific portion of chicken Ku70. Our data show that the chicken *Ku70* gene encodes 614 amino acids. Additionally, our findings indicated that Ku70 localizes in the nucleus as a heterodimer with Ku80 and accumulates at DNA damage sites immediately after injury in chicken cells. Notably, the chromosome 1 genome, housing the chicken *Ku70* gene, does not contain most of the 5′ flanking sequence for chicken *Ku70,* known as *GdKu70* and gained worldwide use. This study provides valuable information and experimental materials to unravel the diverse mechanisms by which Ku functions. Meanwhile, caution is advised when in interpreting the numerous important discoveries made using *GdKu70* cDNA and its expressed cells.

## Results

### The 5′ flanking nucleotide sequences corresponding to the first 18-amino acid coding portion of the reported chicken *Ku70* cDNA cannot be detected at RNA level

**Figure 1:**
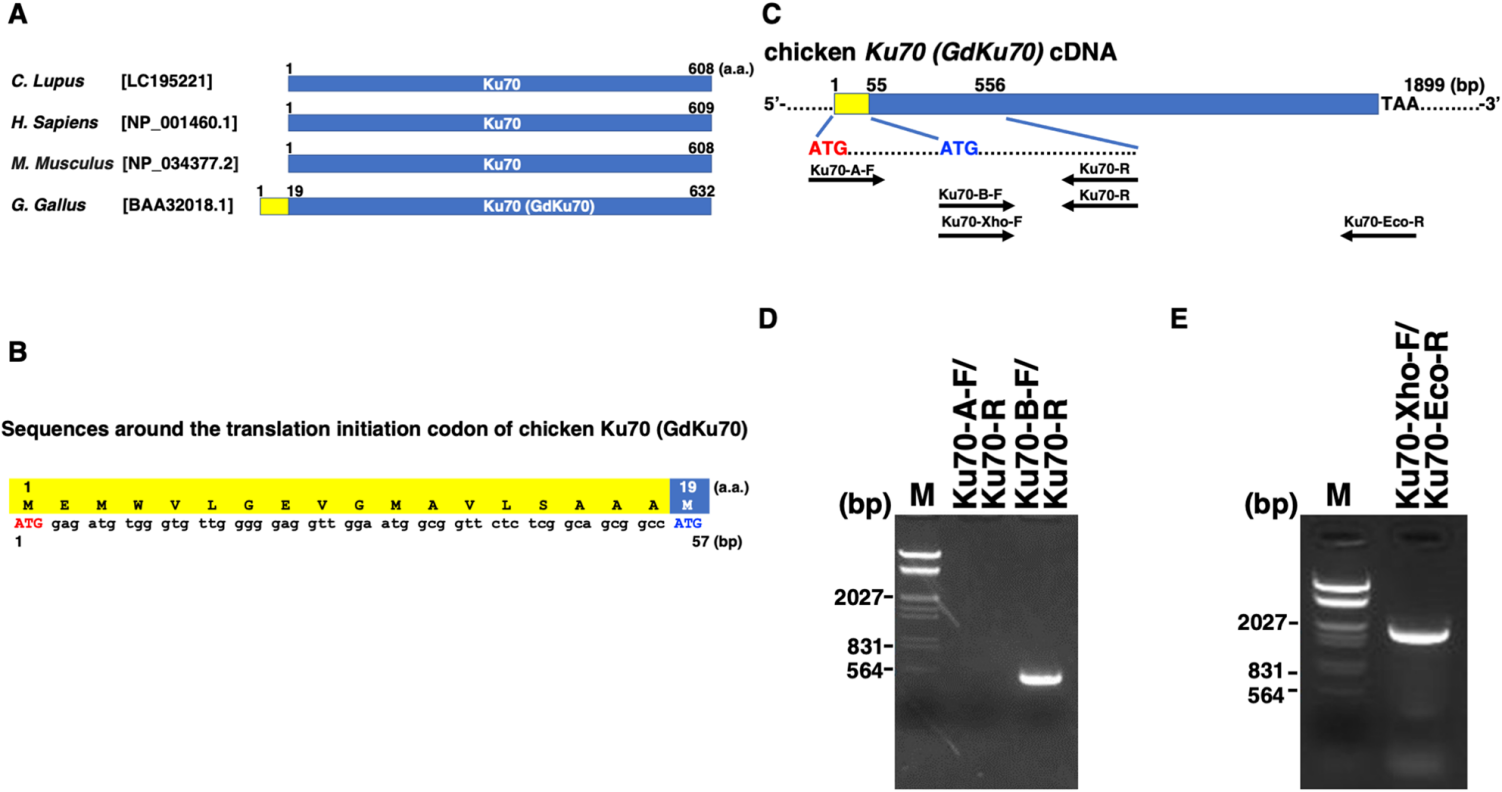
(**A**) Chicken Ku70 (GdKu70) differs from mammalian Ku70 by having an additional 18 amino acids at the N-terminal end. A comparison of Ku70 sequences among chicken (*Gallus gallus domesticus,* GenBank accession No. [BAA32018.1])^5^, dogs (*Canis lupus familiaris*, GenBank accession No. [LC195221])^25^, humans (*Homo sapiens,* GenBank accession No. [NP_001460.1]), and mice (*Mus musculus*, GenBank accession No. [NP_034377.2]). (**B**) Sequences surrounding the chicken Ku70 (GdKu70) translation initiation codon as reported by Takata et al.^5^. An 18-amino acid stretch is appended to the N-terminus in the amino acid sequence of chicken Ku70 (GdKu70) compared to human and mouse Ku70 (highlighted in yellow in **A** and **B**). The reported translation initiation codon and the translation initiation codon predicted by comparison among mammals are shown in red and blue capital letters, respectively. Numbers indicate amino acids (top) and nucleotides (bottom). (**C**) Primers for the detection of chicken *Ku70* by RT-PCR analysis. Specific primer locations used for detecting chicken *Ku70* (*GdKu70*) are indicated. ATG (red capital) is the reported translation initiation codon, and ATG (blue capital) is the predicted translation initiation codon based on comparison with mammals. (**D**) RT-PCR analysis of chicken *Ku70* using two 5′ end primers containing the reported (Ku70-A-F) or predicted translation initiation codons (Ku70-B-F)^5^. The primer pairs (Ku70-A-F/Ku70-R and Ku70-B-F/Ku70-R) were used to amplify each DNA fragment. (**E**) RT-PCR analysis of the chicken *Ku70* coding sequence (CDS). The CDS of *Ku70* was amplified using high-fidelity Platinum™ SuperFi™ DNA Polymerase with the Ku70-Xho-F/Ku70-Eco-R primer set. The amplified DNA was analyzed by 1% agarose gel electrophoresis. M: Lambda phage DNA EcoRI/HindIII digestion marker.

Chicken Ku70 (*GdKu70*) cDNA has previously been cloned and extensively utilized for research^6,7,9–13^. GdKu70 possesses an additional 18-amino acid extension at its N-terminus compared to human and mouse Ku70, although its biological significance remains uncertain^5^. To validate this, we compared the number of amino acids comprising Ku70 among chicken, human, mouse, and canine Ku70. Sequence alignment analyses using published data confirmed that GdKu70 harbors an additional 18 amino acids at its N-terminus compared to Ku70 in three mammalian species, including canine Ku70 (Fig. 1A). These results suggest the possibility that the 18-amino acid segment may have specific functions in chicken and bird cells, despite the absence of major specific functional domains within this region (Fig. 1B). To test this hypothesis, we initially analyzed the expression of chicken *Ku70* mRNA through RT-PCR. Specific primers designed for analyzing the chicken *Ku70* expression were based on a sequence previously identified as *GdKu70*^5^ (Fig. 1C). Subsequently, RT-PCR analysis was conducted using RNA from chicken Leghorn male hepatoma (LMH) cells. This involved using 5′ end primers containing the *GdKu70* specific translation initiation codon (Ku70-A-F) or the predicted translation initiation codon in homologous positions with those of mammalian species (Ku70-B-F), along with a 3′ end primer (Ku70-R) (Fig. 1D). PCR products of the expected length were detected with the primer set Ku70-B-F/Ku70-R, but not with Ku70-A-F/Ku70-R. Additionally, we observed PCR products of the expected length using 5′ end primers (Ku70-Xho-F) and a 3′ end primer (Ku70-Eco-R) (Fig. 1E). These results imply that the 18-amino acid portion may not be expressed in the examined chicken cell line.

**Figure 2:**
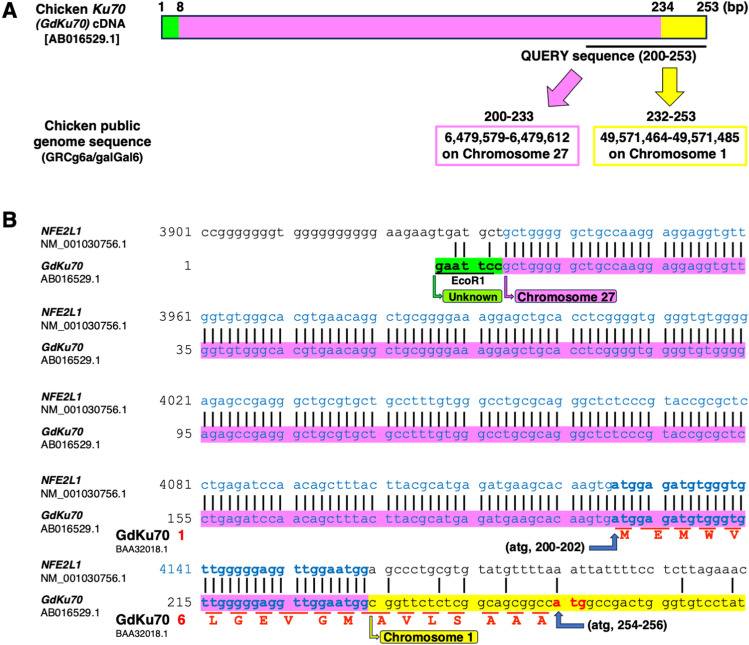
Chicken *Ku70* (*GdKu70*) is a fusion product of three genes derived from chromosomes 1, 27, and an unknown chromosome. (**A**) A nucleotide sequence (54 [bases 200–253] bp in *GdKu70* [AB016529.1]) corresponding to the N-terminal sequence, which displays an 18-amino acid protrusion unique to chickens compared to humans, mice, and dogs, when aligned with the amino acid sequence of Ku70, was subjected to a genomic search against the chicken public genome sequence GRCg6a/galGal6 using the BLAT alignment tool. The results revealed that the QUERY sequence 200–233 perfectly matches with 6,479,579–6,479,612 of chromosome 27, and 232–253 perfectly matches with 49,571,464–49,571,485 of chromosome 1, respectively. (**B**) A homology search was conducted on a nucleotide sequence (274 [bases 1–274] bp in *GdKu70* [AB016529.1]) using BLAT and another alignment tool, BLAST. The sequences included the reported translation initiation codon (atg, 200–202) and the predicted translation initiation codon (atg, 254–256, bold red letters), based on a comparison among mammals. The results suggest that chicken *Ku70* (*GdKu70*) is a fusion product derived from three genes, i.e., an unknown gene including the EcoR1 recognition sequence (underlined) on the unknown chromosome (bases 1–7, green), the *NFE2L1* gene [NM_001030756.1] on chromosome 27 (bases 8–233, pink), and the *GdKu70* gene on chromosome 1 (bases 234–274, yellow). The 18 amino acids in GdKu70 are indicated by red capital letters [BAA32018.1]^5^.

To confirm the genome sequence, a cDNA sequence (54 bp; 200–253 [AB016529.1] and 1–54 [NM_204927.2]) corresponding to the N-terminal sequence, which displayed an 18-amino acid segment unique to chickens compared to humans, mice, and dogs, was subjected to a genomic search against the chicken public genome sequence GRCg6a/galGal6 using the Basic Local Alignment Search Tool (BLAST)-like Alignment Tool (BLAT; https://genome.ucsc.edu/cgi-bin/hgBlat) (UCSC) (Fig. 2A). The results indicated that the QUERY sequence 200–233 matched perfectly with 6,479,579–6,479,612 on chromosomes 27, and 232–253 matched with 49,571,464–49,571,485 on chromosome 1, respectively. Note that residues 232 and 233 in the QUERY sequence overlap because they are both G sequences. As there is no ATG sequence in the QUERY sequence from 232 to 253, we propose that this sequence is situated in the 5′-untranslated region (UTR) of chicken *Ku70* with translation initiated from position 254 to 256 (atg), as deduced from the alignment analysis of human and mouse *Ku70* (Fig. 1A,B). Similar to the findings of Takata et al. (1998), our analysis confirmed that the sequence gccatgg, including this ATG sequence, corresponds to the optimal Kozac sequence, a consensus sequence located around the translation start site^5^. Furthermore, a homology search encompassing the sequence of *GdKu70* from 1 to 274, including the sequences encoding the 18 amino acids, was conducted using BLAT and BLAST. The results suggested the possibility that *GdKu70* results from the fusion of three genes originating from two chicken chromosomes and an unknown chromosome (Fig. 2B). Specifically, this fusion involves the sequence of unknown origin including EcoR1 recognition sequences, the sequence in the 3′-UTR of *Gallus gallus* nuclear factor, erythroid 2 like 1 (*NFE2L1*) [NM_001030756.1] on chromosome 27, and the sequence of chicken *Ku70* on chromosome 1. Of the 18 amino acids previously reported as specific to GdKu70, 11 amino acids are considered to be derived from the sequence of the 3′-UTR of *NFE2L1*. The remaining seven amino acids originate from the 5′-UTR of chicken *Ku70*.

**Figure 3:**
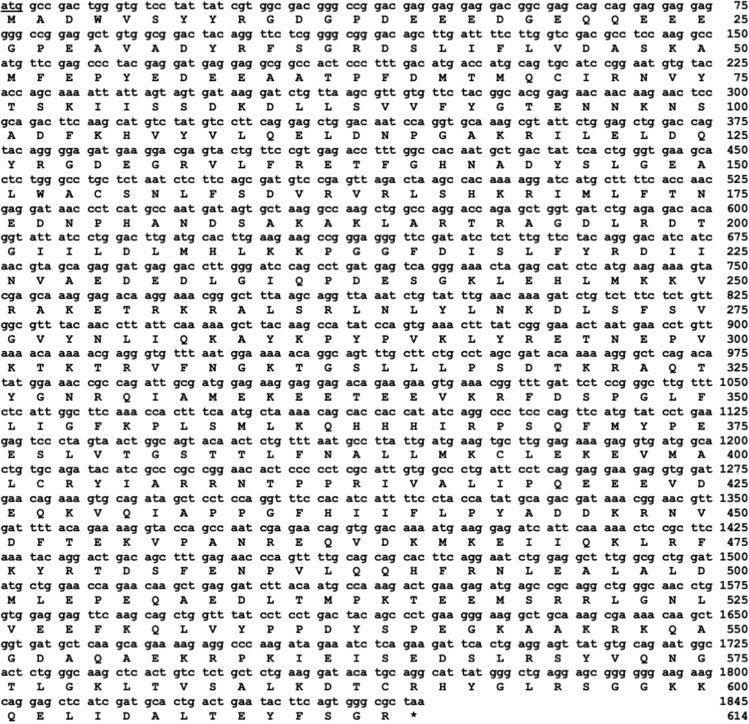
Nucleotide and deduced amino acid sequences of chicken *Ku70* cDNA cloned in this study. The coding sequence of chicken *Ku70* consists of 1845 bp, encoding 614 amino acid residues. The data has been deposited in the DDBJ, EMBL, and GenBank with the accession number [LC750713]. The translation initiation codon (atg) is underlined, and the asterisk indicates the position of the termination codon (taa).

Next, we proceeded to clone and sequence chicken *Ku70* cDNA, encompassing an open reading frame, using 5′ end primers (Ku70-Xho-F) and a 3′ end primer (Ku70-Eco-R) (Fig. 1C,E). As illustrated in Fig. 3, we successfully isolated an 1,845-nucleotide open reading frame encoding the chicken Ku70 protein (614 amino acids). This valuable information has been deposited in the DDBJ/EMBL/NCBI database under the accession number LC750713.

### Comparative analysis of amino acid sequences between chicken Ku70 and mammalian Ku70

**Figure 4:**
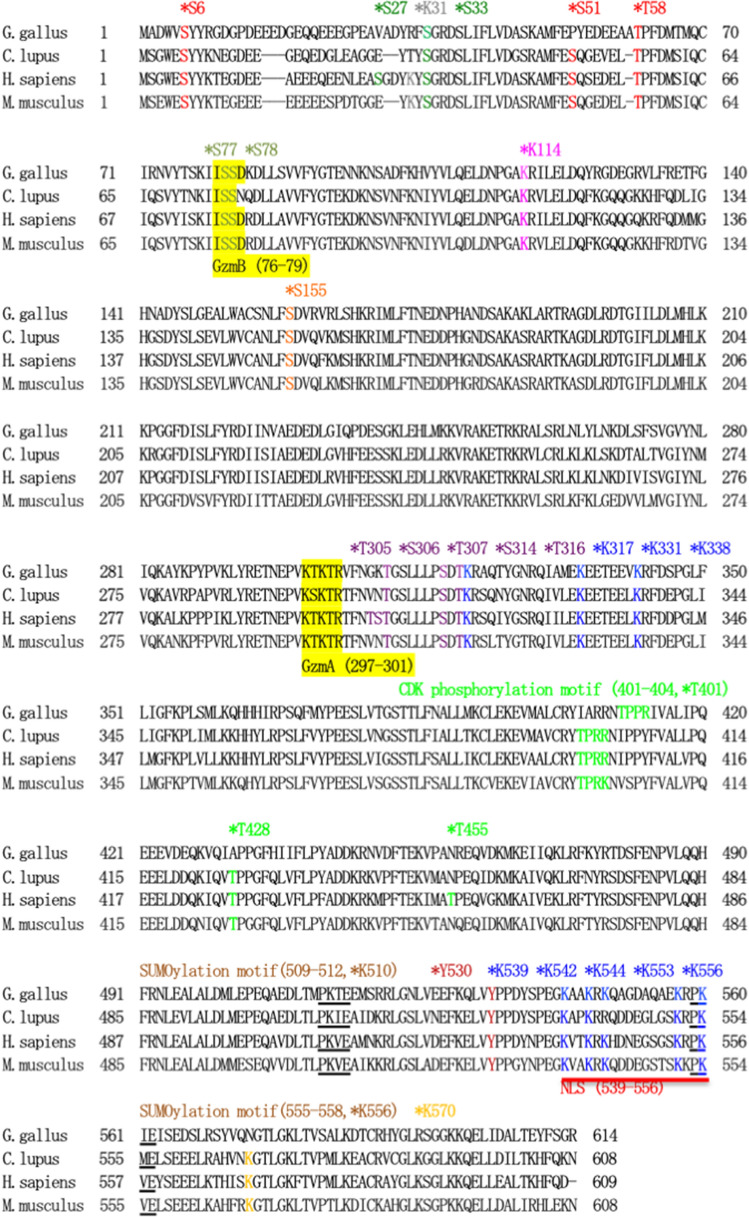
Ku70 sequence alignment. The amino acid sequences of chicken Ku70 (*Gallus gallus domesticus*, [LC750713]) are aligned with those of dog (*Canis lupus familiaris,* [LC195221]), human (*Homo sapiens*, [NP_001460.1], and mouse (*Mus musculus*, [NP_034377.2]) Ku70. The location of the nuclear localization signal (NLS) sequence (NLS: 539–556), the Granzyme A (GzmA) target motif (KTKTR301), the Granzyme B (GzmB) target motif (ISSD79), the two canonical SUMOylation consensus motifs (ψ-K-X-E: 509PKVE512 and 555PKVE558), and the CDK phosphorylation motif ([S/T]Px[K/R]: 401TPRR404) in human Ku70 are shown^25,33–36,41^. Additionally, the location of the candidate nucleophile needed for 5′dRP/AP lyase activity (K31), the DNA-PK phosphorylation sites (S6 and S51), the DNA damage inducible phosphorylation sites (S27, S33, and S155), the phosphorylation sites needed for Ku’s dissociation from DSB (T305, S306, T307, S314 and T316), the PKC α phosphorylation sites (S77 and S78), the Src phosphorylation site (Y530), the cyclin B1/CDK1 phosphorylation sites (T401 and T428), the cyclin A2/CDK2 phosphorylation sites (T401, T428, and T455), the cyclin E1/CDK2 phosphorylation site (T58), the ubiquitination site (K114), the acetylation sites (K317, K331, K338, K539, K542, K544, K553, and K556), the methylation site (K570), and the two SUMOylation sites (K510 and K556) in human Ku70 are marked with asterisks or underlined^25,35,37–49^.

To assess the amino acid sequence of chicken Ku70 in comparison to mammalian Ku70, a comparative analysis was conducted. Chicken Ku70 exhibits a shared amino acid identity (similarity) of 69.4% (84.4%), 69.5% (84.0%), and 66.9% (82.9%) with human, canine, and mouse Ku70, respectively (Supplementary Table S1). In contrast, mouse Ku70 displays a higher amino acid identity (similarity) of 83.1% (90.7%) and 84.0% (90.8%) when compared to human and canine Ku70, respectively. Ku70 is a multifunctional protein, and its functions are potentially regulated, at least in part, by post-translational modifications (PTMs) in human cells, including acetylation, phosphorylation, SUMOylation, protein cleavage, and protein–protein interactions^15,16,25,30–34^. To investigate whether functional domains and modification sites are evolutionarily conserved in chicken Ku70, we compared the amino acid sequence of chicken Ku70 with that of other mammalian species (Fig. 4). Our analysis revealed that certain critical features, including a nuclear localization signal (NLS; 539–556), two SUMOylation consensus motifs (ψ-K-X-E: 509PKVE512 and 555PKVE558), and a protein cleavage motif (Granzyme A [GzmA] target motif [KTKTR301]), presented in human Ku70, are also conserved in chicken, canine, and mouse species. Additionally, another protein cleavage motif (Granzyme B [GzmB] target motif [ISSD79]), found in human Ku70, is conserved in chickens and mice but not in canines^25,33–36^. However, the Lys31 (K31), which is essential for 5′dRP/AP lyase activity in humans, is not conserved in chickens, suggesting that chicken Ku and canine Ku may lack this activity^25,37^. Furthermore, experiments conducted in human and mouse cells have demonstrated that Ku70 function is partially regulated by phosphorylation by kinases activated in a cell cycle-dependent manner, as well as by kinases associated with DNA repair and apoptosis modulation^25,38–42^. Human Ku70, for instance, serves as a substrate for the tyrosine kinase Src, contributing to Ku70-dependent inhibition of apoptosis through phosphorylation of Ku70 at Tyr530 (Y530)^43^. Recent studies have indicated that human Ku70 is phosphorylated by PKC-α at Ser77/78 (S77/S78), which, in turn, suppresses Ku70 binding to DSBs^44^. Importantly, these phosphorylation sites are conserved across all the examined species. Fourthermore, we found that the CDK phosphorylation motif ([S/T]Px[K/R]: 401TPRR404), which is a target for cyclin B1/CDK1 and cyclin A2/CDK2, the putative cyclin E1/CDK2 phosphorylation site (T58), the DNA damage-induced phosphorylation sites (S33 and S155), and putative phosphorylation sites required for Ku70/Ku80 dissociation from DSBs (T307, S314 and T316) in human Ku70 are conserved in chicken, canine, and mouse species^25,38–42^. However, we observed that some phosphorylation sites in human Ku70, including the cyclin B1/CDK1 phosphorylation site (T428), DNA damage-induced phosphorylation site (S27), putative phosphorylation sites needed for Ku70/Ku80 dissociation from DSBs (T305 and S306), and cyclin A2/CDK2 phosphorylation sites (T428 and T455), are not conserved in chickens. Furthermore, among these phosphorylation sites, amino acid residues other than T428 are not conserved in chicken, canine, and mouse species. The two DNA-PK phosphorylation sites (S6 and S51) in human Ku70 are perfectly conserved in canine and mouse species, while S51 is not conserved in chicken^25,45,46^. Additionally, eight acetylation sites (K317, K331, K338, K539, K542, K544, K553, and K556) present in humans are also perfectly conserved in chickens and mice. However, the acetylation site K544 is not conserved in canine species^25,47^. Furthermore, the ubiquitination site (K114) in humans is evolutionarily conserved in chicken, canine and mouse species^25,48^. Finally, it is worth noting that human Ku70 was reported to be methylated at K570 by SET-domain-containing protein 4 (SETD4), with Ku70 methylation being crucial for Ku70 localization to the cytoplasm and subsequent inhibition of apoptosis^49^. However, this methylation event is conserved in all examined mammalian species but not in chickens.

### Subcellular localization of chicken Ku70

**Figure 5:**
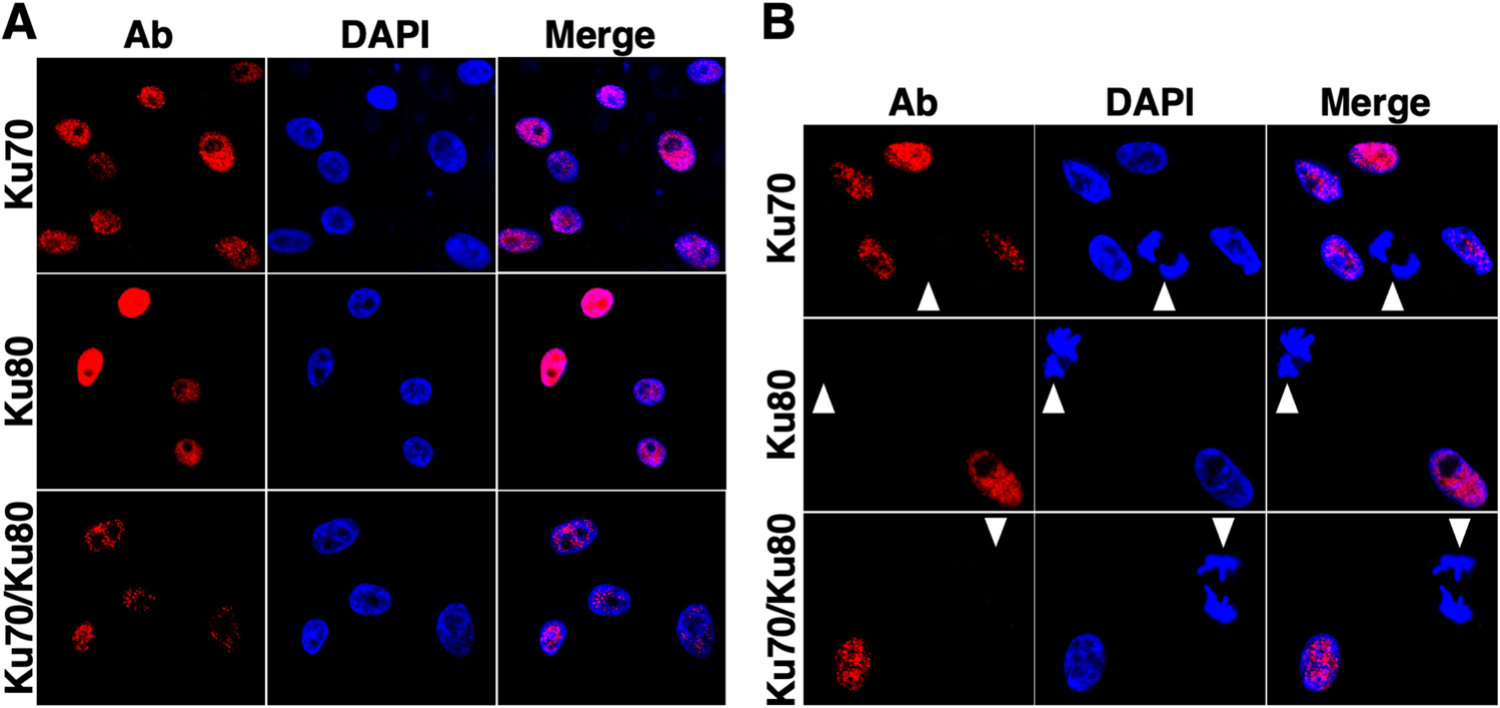
Expression and subcellular localization of Ku70 and its heterodimeric partner, Ku80, in chicken cells. (**A**,**B**) Subcellular localization of Ku70 and Ku80 in chicken PGC29-fibro cells. Cells were fixed and stained with anti-Ku70, anti-Ku80, or Ku70/Ku80 antibodies. Nuclear DNA was stained with DAPI. The stained cells were analyzed using confocal laser scanning microscopy. Cells in interphase and mitotic phase were discriminated using the morphology of cell nuclei visualized by DAPI staining as an indicator. (**A**) Nuclear localization of Ku70 and Ku80 in interphase cells. Left panel: Ku70 (upper), Ku80 (middle), and Ku70/Ku80 (lower) images; center panel: DAPI images; right panel: merged images. (**B**) Low Ku70 and Ku80 expression in mitotic cells. Left panel: Ku70 (upper), Ku80 (middle), and Ku70/Ku80 (lower) images; center panel: DAPI images; right panel: merged images. Arrowheads indicate cells in the mitotic phase.

To examine the subcellular localization of Ku70 in normal chicken cells, we conducted immunofluorescence analysis using confocal laser scanning microscopy (CLSM) in PGC29-fibro cells, a primordial germ cell (PGC) line derived from normal White Leghorn chickens. As shown in Fig. 5A, indirect immunofluorescence staining with the Ku70 antibody revealed fluorescence in the nucleoplasm while excluding the nucleolus in interphase cells. A similar nuclear staining pattern was observed when using the Ku80 antibody, indicating that Ku70 co-localizes with its heterodimeric partner Ku80 within the nuclei of chicken cells. To further corroborate this findings, we performed indirect immunofluorescence staining using an anti-Ku70/Ku80 antibody, which yielded consistent results with individual Ku70 and Ku80 antibodies. Notably, mitotic phase cells did not exhibit detectable fluorescence with any of the three antibodies (Fig. 5B), suggesting that the expression of chicken Ku70 and Ku80 may be lower in mitotic cells compared to interphase cells.

**Figure 6:**
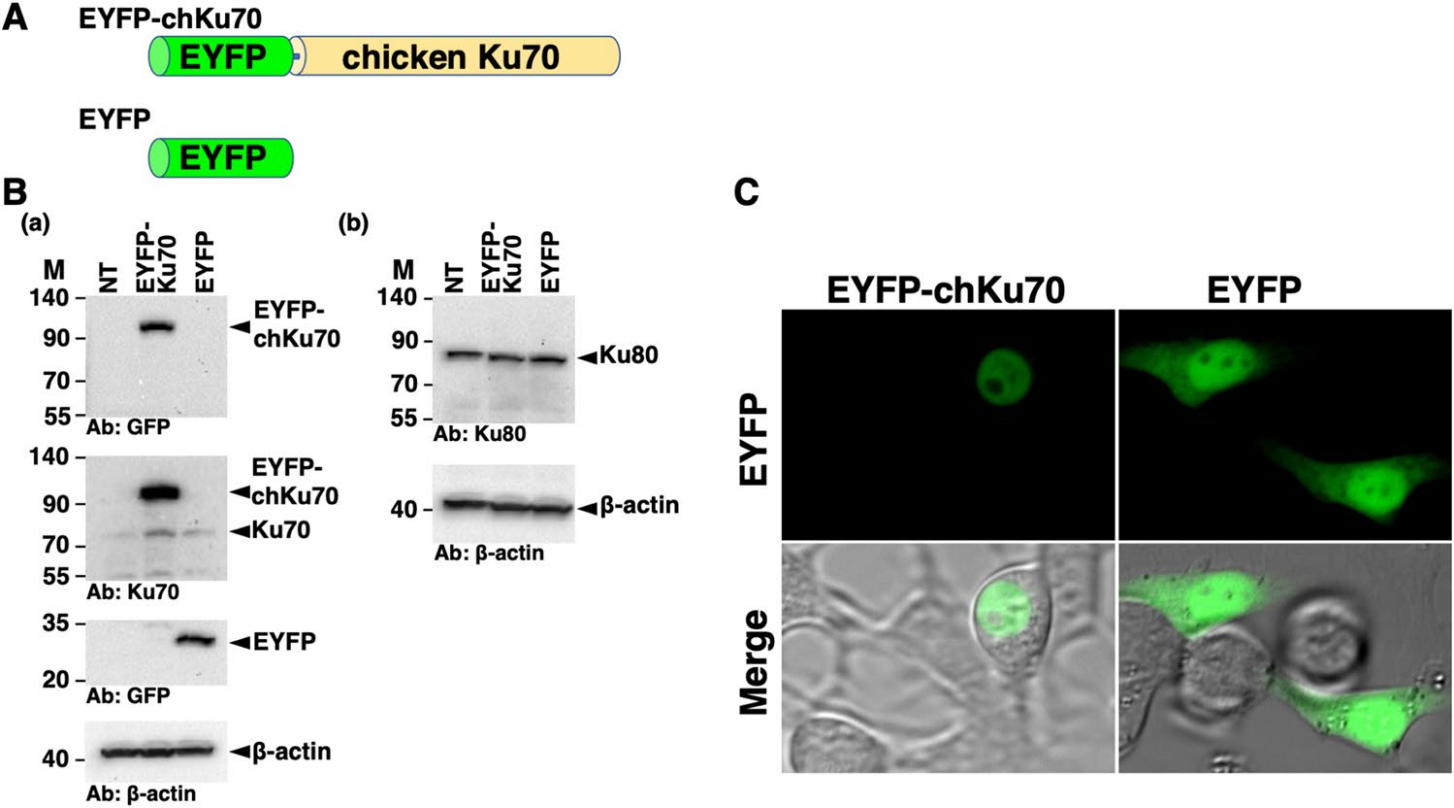
Expression and subcellular localization of EYFP-chicken Ku70 in chicken living cells. (**A**) Scheme representing the EYFP-chicken Ku70 chimeric protein (EYFP-chKu70) and control protein (EYFP). (**B**) (**a**,**b**) EYFP-chKu70 expression in chicken LMH cells. Extracts from cells transiently expressing EYFP-chKu70 or EYFP were analyzed by western blotting using anti-GFP (**a**, top and second from bottom), anti-Ku70 (**a**, second from top), anti-Ku80 (**b**, top), and anti-β-actin (**a**,**b**, bottom) antibodies. NT, non-transfected cells. M, molecular weight marker (kDa). (**C**) Subcellular localization of EYFP-chKu70 in living chicken LMH cells. Cells transiently expressing EYFP-chKu70 or EYFP were examined using confocal laser microscopy. EYFP images of the same cells are shown alone (upper panel) or merged with corresponding differential interference contrast (DIC) (lower panel) images.

To confirm the nuclear localization of chicken Ku70 expressed from the cloned *Ku70* cDNA in live chicken cells, we examined chicken LMH cells transiently expressing EYFP-chicken Ku70 or EYFP alone. Initially, the expression vectors pEYFP-*chicken Ku70* or pEYFP were transfected into the cells (Fig. 6A). Western blot analysis using anti-Ku70 and anti-GFP antibodies confirmed the expression of EYFP-chicken Ku70 in transfected cells (Fig. 6B). Additionally, we detected the expression of chicken Ku80 using an anti-Ku80 antibody. As shown in Fig. 6C, CLSM revealed that EYFP-chicken Ku70 is localized to the nucleoplasm in interphase cells, excluding the nucleolus. In contrast, in pEYFP-transfected cells, EYFP alone was distributed throughout the cell, except in the nucleolus. Collectively, our findings indicate that chicken Ku70 expressed from the cloned cDNA in this study localizes to the nucleus in interphase chicken cells.

### Recruitment of chicken Ku70 to DNA DSB sites

**Figure 7:**
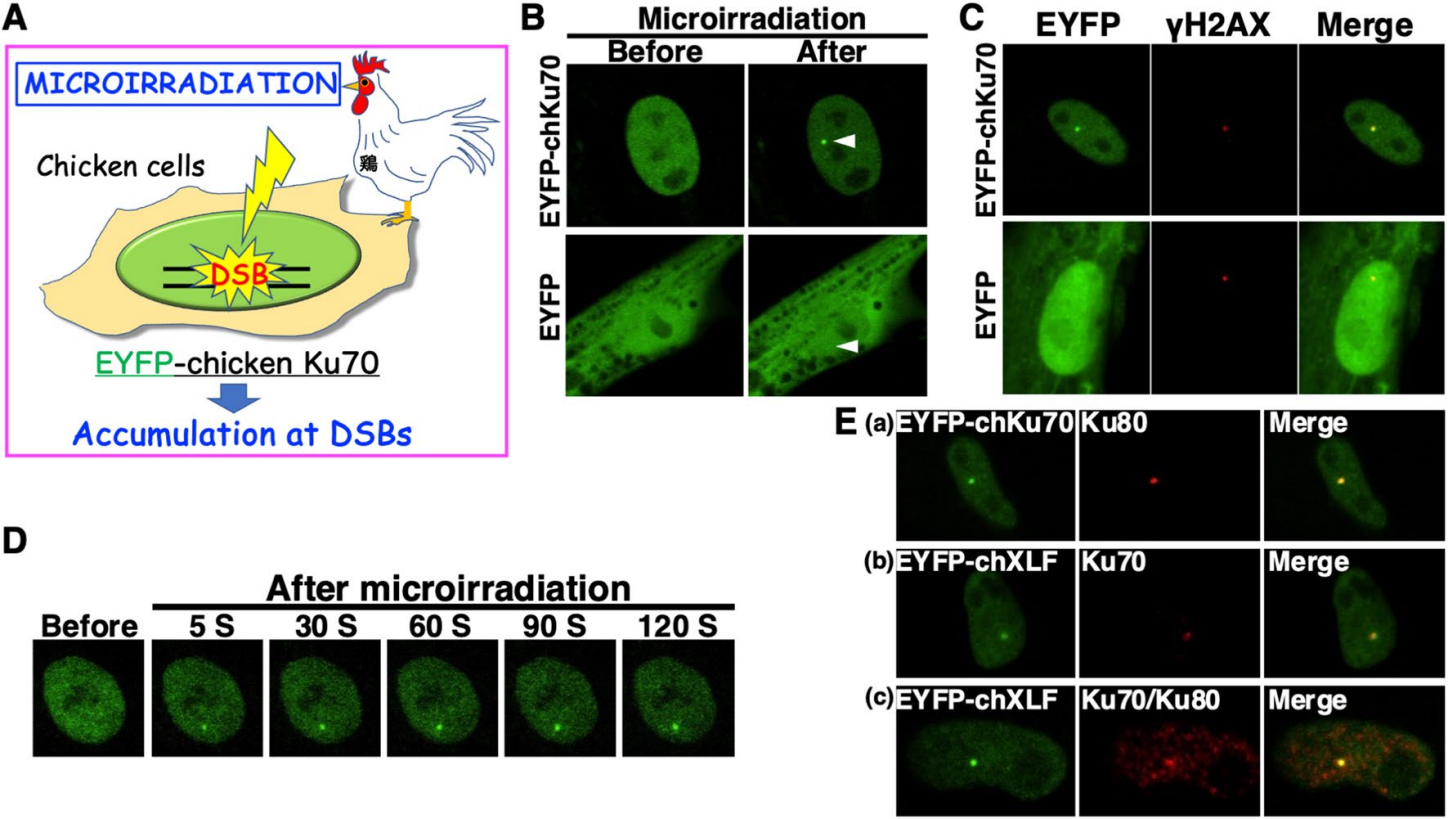
Accumulation of Ku70 to DSBs induced by laser microirradiation in chicken cells. (**A**) The localization and accumulation of EYFP-chicken Ku70 (EYFP-chKu70) at DSBs induced by 405-nm laser irradiation were examined in chicken cells. (**B**) Recruitment of EYFP-chKu70 to the microirradiated site induced by 405-nm laser irradiation in chicken cells. Imaging of live PGC29-fibro cells transfected with pEYFP-*chKu70* (upper panel) or pEYFP (lower panel) before (left panel) and after (right panel) microirradiation. Arrowheads indicate microirradiated sites. (**C**) Accumulation of EYFP-chKu70 at DSBs induced by laser microirradiation in PGC29-fibro cells. Immunostaining of microirradiated cells transfected with pEYFP-*chKu70* (upper panel) or pEYFP (lower panel) was performed using anti-γH2AX antibody 5 min post-irradiation. Left panel, EYFP-chKu70 (upper) or EYFP (lower); center panel, γH2AX; right panel, merged images. (**D**) Time-dependent EYFP-chKu70 accumulation in live PGC29-fibro cells, from 5 to 120 s after irradiation. (**E**) Immunostaining of microirradiated cells transfected with pEYFP-*chKu70* or pEYFP-*chXLF* using anti-Ku80, anti-Ku70, or anti-Ku70/Ku80 antibodies. The cells were fixed and stained with each antibody 5 min post-irradiation. Left panel, EYFP-chKu70 (**a**); EYFP-chXLF (**b** and **c**); center panel, Ku80 (**a**), Ku70 (**b**), and Ku70/Ku80 (**c**); right panel, merged images (**a–c**).

The subcellular localization and accumulation of Ku70 and other NHEJ proteins at DNA DSBs have been well-studied in mammalian cells but not in chicken cells^16,18,25,36,44,49–55^. Additionally, the subcellular localization of DNA repair proteins involved in the NHEJ pathway and their changes after DNA damage have not been reported in avian cells. Recently, we demonstrated that chicken XLF localizes to the nucleus and accumulates at DSBs in chicken cells, while truncated XLF lacking the predicted Ku-binding motif dose not localize to DSBs^53,56^. To ascertain whether chicken Ku70 accumulates at DSBs, we locally induced DSBs in PGC29-fibro cells expressing EYFP-chicken Ku70 or EYFP alone using a 405 nm laser (Fig. 7A). As illustrated in Fig. 7B, EYFP-chicken Ku70, but not EYFP alone, accumulated at the laser-microirradiated sites in live chicken cells during interphase. Microirradiation, coupled with immunostaining against γH2AX, a marker for DSB detection, revealed that EYFP-chicken Ku70 co-localized with γH2AX at microirradiated sites in chicken cells (Fig. 7C). Time-lapse imaging demonstrated that EYFP-chicken Ku70 began to accumulate at DSB sites within 5 s of irradiation (Fig. 7D). Furthermore, microirradiation combined with immunostaining for Ku80 showed that EYFP-chicken Ku70 accumulated and co-localized with Ku80 at DSBs in chicken cells (Fig. 7Ea). Additionally, we observed that the NHEJ repair protein XLF accumulated and co-localized with Ku, a heterodimer composed of Ku70/Ku80, at DSBs (Fig. 7Eb,c). These findings suggest that the NHEJ repair protein Ku70 forms a heterodimer with Ku80 in chicken cells, and Ku, along with other NHEJ repair proteins, such as XLF, may participate in DSB repair immediately after DNA damage. These results also highlight the utility of the chicken Ku70 cloned in this study for investigating the molecular mechanisms of DNA repair in chicken cells.

## Discussion

Chicken *Ku70* cDNA, known as *GdKu70,* and cell lines expressing it have been instrumental in various studies aiming to elucidate Ku70's function and its involvement in Ku70-dependent molecular mechanisms, including the NHEJ pathway and radiation/drug resistance^5–13^. These investigations have led to significant discoveries, such as the selection mechanism of the DNA DSB repair pathway^5^. However, our findings in this study strongly suggest that *GdKu70* cDNA encodes an artificial Ku70 variant (GdKu70) and that GdKu70 is not expressed in chicken cells. Consequently, caution should be exercised when interpreting certain prior results obtained using *GdKu70* cDNA and its expressing cells.

In this study, we conducted a new cDNA cloning of chicken *Ku70*, revealing that it consists of 614 amino acids. It is worth noting that Takata et al*.* previously reported that chicken *Ku70* encodes 632 amino acids, including an 18-amino acid portion specific to chicken Ku70^5^. However, our RT-PCR analysis suggests that *GdKu70* mRNA is not expressed in chicken cells. In a prior study, we demonstrated that chicken *Ku70* is localized on chromosome 1 using direct R-banding fluorescence in situ hybridization^28^. In our current study, genome information analysis utilizing the publicly available genome sequence, revealed that 233 bases, which nearly encompass the chicken-specific 18-amino acid portion in GdKu70, perfectly match a section of the 3′-UTR of *NFE2L1* on chromosome 27. Taken together, it appears that the *GdKu70* cDNA reported by Takata et al. is likely an artificial gene, in which 233 bases from the *NFE2L1* on chromosome 27 have fused to the 5′-UTR, corresponding to the region of chicken *Ku70* on chromosome 1. We also noted an unidentified region, including the EcoR1 recognition sequence, in the 5′-flanking of *GdKu70*^5^. In conclusion, the portion of chicken-specific 18 amino acids previously reported does not appear to be expressed in chicken cells. It is worth mentioning that Takata et al*.* (1998) reported isolating *Ku70* full-length cDNA and genomic DNA, which codes for GdKu70 from a chicken intestinal mucosa cDNA library (Clontech) and a liver genomic library (Stratagene), respectively^5^. Moreover, the identities of the clones were confirmed through sequencing. Nevertheless, it remains unclear why the same fusion occurred in the cDNA and genomic libraries derived from different organs and purchased from different companies.

The multifunctional protein Ku70 plays critical roles not only in NHEJ but also in various mechanisms, including V(D)J recombination, telomere maintenance, and regulation of neuronal apoptosis^15,16,30–32^. Understanding the regulation of Ku70's subcellular localization is crucial for comprehending its function, although this regulatory mechanism remains incompletely understood^16^. Our findings indicate that chicken Ku70 predominantly localizes in the cell nucleus during interphase. Furthermore, the heterodimer Ku, composed of Ku70 and Ku80, accumulates at DNA DSBs immediately after DNA damage in chicken cells. These observations strongly suggest that the subcellular localization and behavior of Ku following DSB damage are akin to those of human, mouse, and canine Ku^18,25,51,52^. The subcellular localization of Ku70 and the mechanisms controling it are important for the functional regulation of Ku70 and Ku in mammalian species, including humans^16,32^. In human cells, the nuclear localization of Ku70 is mainly regulated by the importin complex binding to the Ku70 NLS and may, in part, depend on the Ku80 NLS through heterodimerization with Ku80^36,57,58^. Recent research has revealed that Ku70's cytoplasmic localization induced by methylation at K570 by SETD4 is essential for regulating Ku70's function in human cells^49^. Furthermore, we reported that acetylation of two lysine residues, K553 and K556, in the human Ku70 NLS can regulate its nuclear localization^59^. In this study, we confirmed that the structure of the human Ku70 NLS is conserved in chicken Ku70, as well as in canine and mouse Ku70. Among the PTM target amino acids involved in regulating the subcellular localization of human Ku70, the acetylation target lysins in the NLS were evolutionarily conserved in chicken Ku70, but the methylation target lysine regulated by SETD4 was not. Notably, our findings showed that both the acetylation target lysins and the methylation target lysine by SETD4 are conserved in the Ku70 of dogs and mice. Overall, our results suggest that while the NLS structure crucial for regulating the subcellular localization of human Ku70 is conserved in chicken Ku70, the regulatory mechanisms of PTMs that negatively modulate NLS-mediated nuclear localization may not be fully conserved in chicken cells. Further studies are needed to elucidate the differences between mammals and birds, including chickens.

Studies involving chickens and chicken cells, such as DT40 cells, have made significant contributions to basic research in the life sciences and pre-clinical research^1–14^. DT40 cells, in paticular, have served as valuable models for studying gene function and have significantly advanced research in life sciences, including DNA repair mechanisms and oncogenesis. Single KO and DKO DT40 cell lines targeting key NHEJ genes, such as *Ku70, XRCC4, XLF, DNA-PKcs,* and *DNA-ligase IV*, have already been generated and studied for their sensitivity to radiation and various drugs^5,6,8,60^. Furthermore, it is now feasible to transplant genetically modified chicken PGCs into recipient embryos to generate genetically modified chick^61^. Detailed investigations using various NHEJ gene-KO DT40 cell lines and new genetically modified chicken models are expected to lead to the discovery of new pathological models resulting from mutations in chicken NHEJ genes and novel regulatory mechanisms related to Ku70 function. Recently, we demonstrated that one chicken breed, the Japanese Bantam (Chabo), has lost a portion of the NHEJ repair gene *XLF*, resulting in decreased DSB repair ability in its cell lines and brain tissue^56^. Furthermore, the pathogenesis in Chabo chickens resembles that of *XLF* deficiency syndrome in humans^22,56^. These findings strongly indicate that chickens serve as valuable analytical models for studying human DNA repair pathways and gene-deficient diseases related to DNA repair. In conclusion, the information and materials obtained in this study are expected to significantly contribute not only to chicken research but also to preclinical and basic life science research in both human and veterinary fields.

## Methods

### Cell lines, cell culture, and transfections

The adherent fibroblast-like cell line PGC29-fibro, derived from PGCs of the White Leghorn line (WL-M/O), was purchased from the NAGOYA UNIVERSITY through the National Bio-Resource Project of the MEXT, Japan. Two chicken cell lines, PGC29-fibro and the LMH cell line (Health Science Research Resources BANK, Osaka, Japan), were cultured in Dulbecco's modified Eagle's medium (Nacalai Tesque, Kyoto, Japan or NISSUI, Tokyo, Japan) supplemented with 60 μg/mL kanamycin and 10% fetal bovine serum. The cells were maintained in a humidified incubator at 37 °C under a 5% CO_2_ atmosphere. Plasmids were transfected into the cells using Lipofectamine 3000 (Thermo Fisher Scientific, Waltham, MA, USA)^55,56^. Following transfection, the cells were cultured for 2 d, and images of the cells were captured using an FV300 CLSM system (Olympus, Tokyo, Japan), as previously described^55,56^.

### RNA preparation, cDNA synthesis, and RT-PCR analysis

RNA was extracted from frozen chicken LMH cells using the RNeasy Mini Kit (QIAGEN Inc., Chatsworth, CA, USA). This RNA served as a template for cDNA synthesis, which was performed using the High-Capacity RNA-to-cDNA™ Kit (Thermo Fisher Scientific). PCR amplification with sense (Ku70-A-F: 5′-atggagatgtgggtgttgggg-3′ or Ku70-B-F: 5′-atggccgactgggtgtcctattatc-3′) and antisense (Ku70-R: 5′-tggcttagtctaactcggacatcgc-3′) primers was performed using the AmpliTaq Gold360 Master Mix (Applied Biosystems, Waltham, MA, USA) according to the manufacturer's instructions. The reaction conditions included initial denaturation at 95 °C for 5 min, followed by 35 cycles of denaturation at 95 °C for 30 s, annealing at 60 °C for 30 s, and extension at 72 °C for 30 s, with a final extension step at 72 °C for 7 min. The amplified DNA was analysed by 1% agarose gel electrophoresis.

### Chicken *Ku70* cloning and expression vector

Oligonucleotide primers designed to amplify chicken *Ku70* cDNA were created to clone the coding sequence of Ku70 in-frame between XhoI and EcoRI sites of pEYFP-C1 (Takara Bio Inc., Shiga, Japan), based on the published *Ku70* cDNA sequence of *G. gallus domesticus* (chicken) (DDBJ/EMBL/GenBank accession No. AB016529.1)^5^. The sense primer (Ku70-Xho-F: 5′-CGCGGAACTCGAGCTatggccgactgggtgtcctattatc-3′) and antisense primer (Ku70-Eco-R: 5′-CCGTGAATTCttagcgcccactgaagtattcagtc-3′) incorporated XhoI and EcoR1 restriction enzyme sites, respectively. High-fidelity Platinum™ SuperFi™ DNA Polymerase (Thermo Fisher Scientific) was used for PCR amplification following the manufacturer's instructions. The PCR conditions included an initial denaturation step at 95 °C for 1 min, followed by 30 cycles of denaturation at 95 °C for 0.5 min, annealing at 60 °C for 0.5 min, and extension at 72 °C for 1 min. The PCR products were digested and ligated in-frame into the pEYFP-C1 vector using the DNA Ligation Kit, Mighty Mix (Takara Bio, Inc.), and the inserts were confirmed by sequencing.

### Western blot analysis

The preparation of total protein extracts and western blot analysis was conducted as previously described^55–57^, with some modifications. Specifically, the total proteins (50 μg per lane) were separated on an Extra PAGE One Precast Gel 5–20% (Nacalai Tesque). Subsequently, the membranes were blocked with Blocking One (Nacalai Tesque) for 30 min at room temperature. The following antibodies were employed: mouse anti-Ku70 monoclonal antibody (E-5, Santa Cruz Biotechnology, Santa Cruz, TX, USA), mouse anti-Ku80 monoclonal antibody (B-4, Santa Cruz Biotechnology), rabbit anti-GFP polyclonal antibody (FL, Santa Cruz Biotechnology), and mouse anti-β-actin monoclonal antibody (AC-15, Sigma-Aldrich, St. Louis, MO, USA). Secondary antibodies used included anti-mouse IgG, horseradish peroxidase (HRP)-linked whole antibody from sheep (GE Healthcare Bio-Sci. Corp., Piscataway, NJ, USA) or anti-rabbit IgG, HRP-linked whole antibody from donkey (GE Healthcare Bio-Sci. Corp.). Protein bands were detected following the manufacturer's instructions using Chemi-Lumi One Ultra (Nacalai Tesque) and visualized with the ChemiDoc XRS System (Bio-Rad, Hercules, CA, USA). The 3-Color prestained XL-ladder (APRO Science, Tokushima, Japan) served as the molecular weight marker.

### DNA damage induction using microlaser and immunofluorescence cell staining

Local DNA damage was induced through a microlaser, and subsequent cell imaging was performed as previously outlined^51,56^. In brief, DSBs were generated locally using a 405 nm diode laser equipped with an FV300 CLSM system (Olympus). Live and fixed cells expressing EYFP-chicken Ku70, EYFP-chicken XLF, or EYFP alone were imaged using an FV300 CLSM system (Olympus). Immunocytochemistry was conducted as previously described^51,55,56^ with the use of the following antibodies: mouse anti-Ku70 monoclonal antibody (E-5, Santa Cruz Biotechnology), mouse anti-Ku80 monoclonal antibody (B-4, Santa Cruz Biotechnology), mouse anti-Ku70/Ku80 monoclonal antibody (162, NeoMarkers, Fremont, CA, USA), mouse anti-γH2AX monoclonal antibody (JBW301, Merck Millipore, Billerica, USA), and donkey anti-mouse IgG (H + L) Highly Cross-Adsorbed Secondary Antibody, Alexa Fluor™ Plus 555 (Thermo Fisher Scientific).

### Sequence analysis

The *GdKu70* cDNA sequence (54 bp [bases 1–54; NM_204927.2] and 54 bp [bases 200–253; AB016529.1]) corresponding to the N-terminal sequence, which displayed an 18-amino acid protrusion unique to chickens compared to humans and mice, was subjected to a genomic search against the chicken public genome sequence GRCg6a/galGal6. This search was performed using the alignment tool BLAT provided by UCSC. Additionally, the homology search encompassing the *GdKu70* cDNA sequence (274 bp [bases 1–274; AB016529.1]), including the sequences encoding the 18 amino acids, was conducted using BLAT and BLAST (https://blast.ncbi.nlm.nih.gov/Blast.cgi). The BLAST was employed to identify regions of local similarity between sequences. The Pairwise Sequence Alignment EMBOSS Needle was utilized to compare the amino acid sequence of chicken Ku70 with those of canine, human, and mouse Ku70 (The European Bioinformatics Institute [EMBL-EBI]; https://www.ebi.ac.uk/Tools/psa/)^62^.

### Supplementary Information

Supplementary Information.

## Data Availability

The sequence of chicken *Ku70* cloned in this study has been deposited in the DDBJ/EMBL/NCBI database under accession number LC750713.
