# Supplementary material for: Molecular cloning, subcellular localization, and rapid recruitment to DNA damage sites of chicken Ku70
Source: Sci Rep. 2024 Jan 12;14:1188. doi: 10.1038/s41598-024-51501-0 (PMC10786929; doi:10.1038/s41598-024-51501-0)
Supplement: Supplementary file 1 — Supplementary Information. [file 41598_2024_51501_MOESM1_ESM.pdf]

Supplementary Information

Molecular cloning, subcellular localization, and rapid recruitment to DNA damage sites of chicken *Ku70*

Manabu Koike[1, 2*], Hideji Yamashita[3], Yasutomo Yutoku[1] & Aki Koike[1]

[1]Institute for Quantum Medical Science, National Institutes for Quantum Science and Technology, 4-9-1 Anagawa, Inage-ku, Chiba 263-8555, Japan.
[2]Life Science Course, Graduate School of Science and Engineering, Saitama University, 255 Shimo-Okubo, Sakura-ku, Saitama, Saitama 338-8570, Japan.
[3]Department of Food and Life Sciences, School of Agriculture, Tokai University, 9-1-1 Toroku, Higashi-ku, Kumamoto 862-8652, Japan.

[*]Corresponding author: koike.manabu@qst.go.jp

Supplementary Figure S1

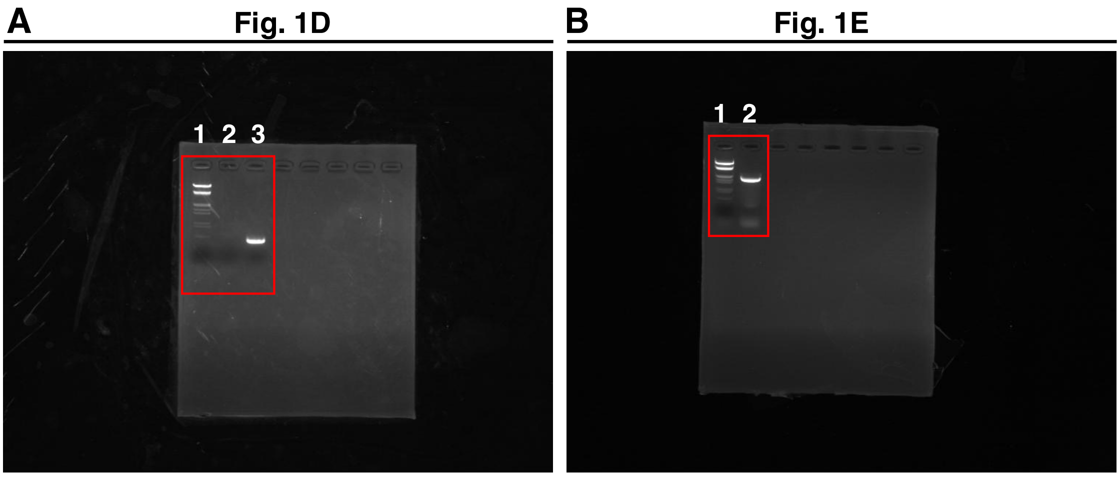

Supplementary Figure S1

(**A**) Uncropped gel image of Fig. 1D. The bands inside the red frame were utilized in this paper. Lambda phage DNA EcoRI/HindIII digestion marker (Lane 1). The primer pairs (Lane 2, Ku70-A-F/Ku70-R; Lane 3, Ku70-B-F/Ku70-R) were employed to amplify each DNA fragment. (**B**) Uncropped gel image of Fig. 1E. The bands inside the red frame were used in this paper. Lambda phage DNA EcoRI/HindIII digestion marker (Lane 1). The CDS of Ku70 was amplified using the Ku70-Xho-F/Ku70-Eco-R primer set. The PCR products were analyzed on a 1% agarose gel.

Supplementary Figure S2

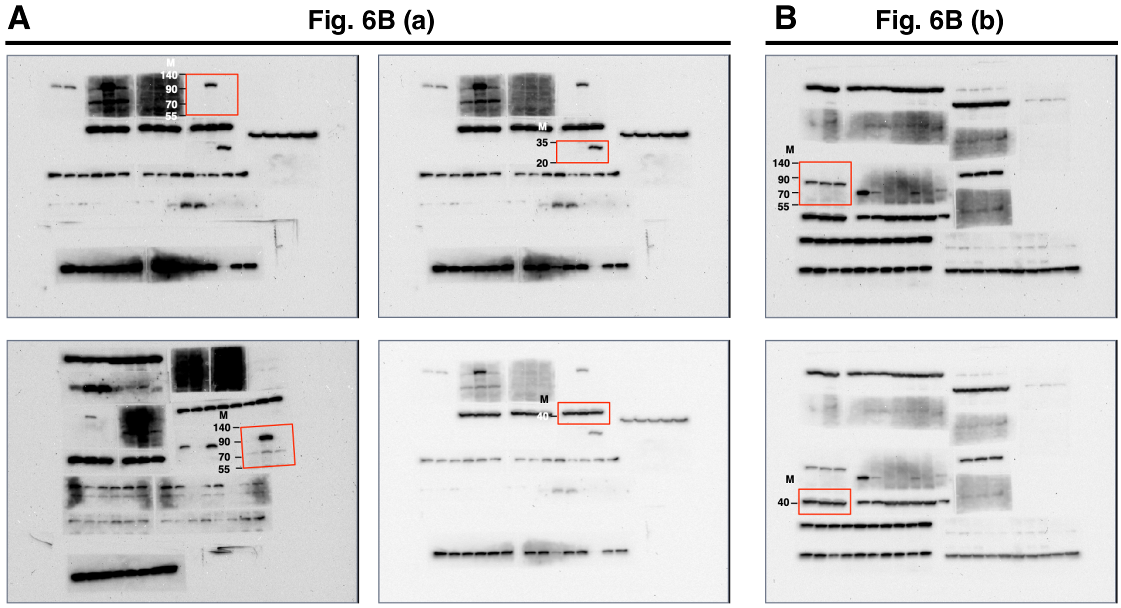

Supplementary Figure S2

(**A**) Raw images of western blots in Fig. 6B (a). The bands inside the red frame were utilized in this paper. GFP (Top left), Ku70 (Bottom left, Re-probing), GFP (Top right), and β-actin (Bottom right). They were detected at exposure times of 6 (Bottom right), 40 (Top right), 90 (Top left), and 150 (Bottom left) minutes, respectively. (**B**) Raw images of western blots in Fig. 6B (b). The bands inside the red frame were employed in this paper. Ku80 (Top) and β-actin (Bottom). They were detected at exposure times of 14 (Bottom) and 60 (Top) minutes, respectively.

Supplementary Table S1

Supplementary Table S1 Comparison of Ku70 amino acid sequences

**Protein** (accession number*)	**Identity with chicken Ku70****	**Similarity to chicken Ku70**	**Identity with mouse Ku70**	**Similarity to mouse Ku70**
**human Ku70** **[NP_001460.1]**	**69.4 %**	**84.4 %**	**83.1 %**	**90.7 %**
**canine Ku70** **[LC195221.1]**	**69.5 %**	**84.0 %**	**84.0 %**	**90.8 %**
**mouse Ku70** **[NP_034377.2]**	**66.9 %**	**82.9 %**	**-**	**-**

* accession number in DDBJ/EMBL/NCBI database, ** chicken Ku70 [LC750713].
